# Apparent autistic traits in transgender people: a prospective study of the impact of gender-affirming hormonal treatment

**DOI:** 10.1007/s40618-022-01835-1

**Published:** 2022-07-02

**Authors:** F. Mazzoli, E. Cassioli, J. Ristori, G. Castellini, E. Rossi, C. Cocchetti, A. Romani, T. Angotti, G. Giovanardi, M. Mosconi, V. Lingiardi, A. M. Speranza, V. Ricca, L. Vignozzi, M. Maggi, A. D. Fisher

**Affiliations:** 1 Andrology, Women’s Endocrinology and Gender Incongruence Unit, Careggi University Hospital, Florence University Hospital, Florence, Italy; 2 Department of Experimental, Clinical and Biomedical Sciences, Careggi University Hospital, University of Florence, Florence, Italy; 3 Psychiatry Unit, Department of Health Sciences, University of Florence, Florence, Italy; 4 Department of Dynamic and Clinic Psychology, Faculty of Medicine and Psychology, Sapienza University of Rome, Rome, Italy; 5 Azienda Ospedaliera San Camillo Forlanini, Rome, Italy

**Keywords:** Gender dysphoria, Autism spectrum disorder, Autistic traits, Gender affirming hormonal therapy, Gender affirming path, Transgender people

## Abstract

**Purpose:**

We evaluated differences in Autism Spectrum Quotient (AQ) scores between a sample of hormone-naïve transgender and cisgender people and the impact of gender-affirming hormonal treatment (GAHT) on AQ scores across time. Furthermore, we assessed alexithymia and social anxiety as possible mediators of changes in AQ scores.

**Methods:**

A cross-sectional comparison between cisgender and transgender people before GAHT and a prospective study on the effects of GAHT over time were performed. Transgender and cisgender people completed several psychometric tests. A total sample of 789 persons (*n* = 229 cismen; *n* = 172 ciswomen; *n* = 206 transmen; *n* = 182 transwomen) referring to the Florence and Rome Gender Clinics between 2018 and 2020 was enrolled. Of these, 62 participants referring to the Florence Gender Clinic were evaluated in a prospective study at baseline and 12 months after GAHT.

**Results:**

Groups showed significant differences in terms of autistic traits: ciswomen showed lower scores of AQ, while cismen reported higher scores of AQ than all other groups. Transgender individuals showed significant higher levels of Gender Dysphoria (GD), body uneasiness, alexithymia and social anxiety, compared to cisgender ones. No significant differences in general psychopathology were found between groups. Across time, transmen and transwomen showed a significant reduction in AQ scores. The decrease in alexithymia and social anxiety after GAHT did not predict the change in AQ scores.

**Conclusions:**

The autistic traits in our sample may represent an epiphenomenon of GD rather than being part of an Autism Spectrum Disorder (ASD) condition, since they significantly decreased after 12 months of GAHT.

## Introduction

In recent years, there has been a strong and growing interest in the co-occurrence of Gender Dysphoria (GD) and Autism Spectrum Disorder (ASD), both in children/adolescents and adults [1–9]. This association is of great clinical interest because of its implications in the management of both conditions. GD is defined in the DSM-5 as the distress resulting from the incongruence between perceived and assigned gender, associated with clinically significant impairment in social, occupational or other important areas of functioning [10]. ASD is a neurodevelopmental condition characterized by deficits in social communication and social interaction across multiple contexts, associated with restricted, repetitive patterns of behaviour and interests [10].

Although these two conditions are relatively rare in the general population, in the literature a series of case-reports [11–18] and studies on their co-occurrence [1, 4–6, 19–22] have been reported. In fact, ASD has a prevalence of 20.6:10.000 (0.21%) in the general population with a male-to-female ratio of 4.2 to 1[23]. GD’s rates range from 1:13.000 to 1:20.000 for assigned males at birth (AMAB) and from 1:3.000 to 1:15.000 for assigned females at birth (AFAB) in the general population [24–26] with an AMAB–AFAB ratio of 1 to 1 [27]. In a systematic review of Zhang and Colleagues (2020) the proportion of people who identify themselves as transgender and gender diverse (TGD) in the general population rates from 0.5% to 4.5% (per 100.000 enrollers) [25]. In 2010 De Vries and Colleagues described a lifetime prevalence of ASD of 7.8% in a sample of children/adolescents referred to a gender clinic [20]. These findings suggest that the over-representation of co-occurring GD and ASD is not the result of chance, but there may be an aetiological link between them.

Some authors speculated that the Extreme Male Brain (EMB) theory plays a role in this co-occurrence [19, 21, 22]. According to this theory [28], individuals with ASD display an extreme version of the typical male brain, where the systemizing domain—the drive to analyse or construct systems—is more developed and the empathizing domain more decreased. Previous studies demonstrated that increased levels of testosterone in amniotic fluid predict autistic cognitive traits in childhood [29, 30]. Moreover, prenatal exposure to androgens in certain conditions may increase GD occurrence in AFAB [31–34]. In line with the EMB theory, Jones and Colleagues (2012) found higher rates of autistic traits in transmen compared to transwomen [21]. This theory explains the higher susceptibility of transmen with ASD to develop GD, but it fails to explain the link between ASD and GD in transwomen. Furthermore, Pasterski and Colleagues (2014) did not find differences in autistic traits between adult transmen and transwomen, in contrast with the EMB theory [22].

Currently, the neurobiological basis of the association between ASD and GD is not yet clear and knowledge on it is far from complete. In particular, there are few data regarding autistic traits in transgender adults, because literature has been mostly focused on children and adolescents. Moreover, prospective studies assessing the effects of gender-affirming hormone therapy (GAHT) on autistic traits are lacking. A recent study of Nobili and Colleagues (2020) aimed to explore the impact of GAHT on autistic traits in a sample of UK transgender adults, reported that autistic traits remained stable after 12 months of GAHT [5]. These results suggest that autistic traits in transgender people seems to be independent from a gender affirming path. This is apparently in contrast with studies that assume that autistic traits may represent an epiphenomenon of GD due to social deficits and minority stress that transgender people may experience [35, 36]. However, these hypothesis have never been demonstrated and required further understanding.

In line with this, the aims of our study are: (i) to evaluate autistic traits in adult transgender persons by assessing differences in terms of AQ scores in a sample of hormone-naïve transgender people and a group of cisgender individuals age-adjusted; (ii) to assess the possible impact of GAHT on AQ scores across time; (iii) to evaluate the role of alexithymia and social anxiety as possible mediators of changes in AQ scores.

## Materials and methods

### Study design

The present study was conducted at the Gender Clinics of the University of Florence and Rome between 2018 and 2020.

For ethical issues, it is not possible to perform a randomized, placebo-controlled study (with a no-GAHT control group); consequently, we performed a double-design study with a cross-sectional comparison between cisgender and transgender people at baseline (before GAHT) and a prospective study on the possible effect of GAHT over time in transgender sample.

The study protocol was approved by the institution’s ethics committee. All of the participants provided written informed consent to participate in the study.

### Cross-sectional study

Persons referring for the first time to the Gender Clinics of the University of Florence and Rome were enrolled in the study, provided they met the following inclusion criteria: age older than 18 years and GD according to the DSM 5 criteria [10]. Furthermore, a control group of age-matched students of the University of Florence and Rome was considered, provided they met the inclusion criteria of age older than 18 years. The exclusion criteria for both groups were: intellectual disability, illiteracy, any kind of hormonal treatment and gender-affirming surgery (GAS).

A total sample of 789 persons (*n* = 229 cismen; *n* = 172 ciswomen; *n* = 206 transmen *n* = 182 transwomen) were included in the study.

### Prospective study

A subsample of participants in the cross-sectional study referring to Florence Gender Clinic was enrolled in a prospective study, provided they asked to start GAHT at the time of inclusion. A total of 62 participants were included before GAHT and evaluated at 12 months (T12) after GAHT prescription. Of the included sample, 24 (39%) were transwomen and 38 (61%) transmen.

All transwomen received oral cyproterone acetate (50 mg) in association with oral estradiol valerate (75%) or transdermal estradiol (25%). All transmen received testosterone undecanoate 1000 mg im, with the first injection repeated after 6 weeks, and then after 12 weeks. The injection interval was adjusted (normally between 10 and 14 weeks) based on serum testosterone levels with the aim of obtaining hormone levels in the normal reference range for males and hematocrit. All patients received psychological support every 3 months.

### Measures

At the time of the first referral to the clinics, sociodemographic data, as well as information about substance abuse and psychiatric medication, were collected through standard questions.

Furthermore, intellectual disability was assessed during the psychological evaluation.

Moreover, transgender and cisgender people were asked to complete several psychometric tests, such as Autism Spectrum Quotient (AQ) [37, 38], Gender Dysphoria Questionnaire for Adolescents and Adults (GIDYQ-AA) [39, 40], Body Uneasiness Test (BUT) [41], Toronto Alexithymia Scale (TAS-20) [42], Liebowitz Social Anxiety Scale (LSAS) [43, 44], and Symptom Checklist 90 revised (SCL-90-R) [45, 46].

The AQ is a self-report 50-items questionnaire evaluating the extent of autistic traits through a 4-point Likert scale, from definitely agree to definitely disagree. It consists of 5 subscales exploring social skills, attention to detail, attention switching, communication and imagination. Higher scores indicate poorer social skills, difficulty in switching attention and in communication with others, stronger attention to detail and tend to lack in imagination (range score 32–50 is suggestive of significant autistic traits) [37, 38].

The GIDYQ‐AA is a self-report questionnaire evaluating GD on a 5‐point response scale (from 1 = always to 5 = never) taking into consideration the past 12 months as time frame. It consists of 27 items which gather different indicators of GD grouped in 4 subscales (subjective GD, social, somatic and socio-legal). Lower scores indicate higher levels of GD [39, 40].

The BUT is a self-rating scale composed of two parts assessing different areas of body-related psychopathology, such as weight phobia, compulsive control behavior, avoidance, experience of strangeness from the body and specific worries about certain body parts or characteristics. Subjects evaluate 34 different experiences with body image (BUT A) and 37 body parts (BUT B) on a 6‐point Likert scale (from 1 = never to 6 = always) and rate how often they happen to dislike each experience or body part. Higher scores indicate greater body uneasiness [41].

The TAS-20 is a 20-items questionnaire evaluating inability or difficulty in experiencing, identifying and communicating emotions. Each item is rated on a 5-point Likert scale (from 1 = completely disagree to 5 = completely agree). Higher scores indicate greater levels of alexithymia [42].

The LSAS is a 24-item questionnaire exploring social anxiety implications in different ordinarily situations through two subscales (fear/anxiety and avoidance) considering the past week as time frame. Each item is first rated on a 4-point Likert scale (from 0 = none to 3 = severe) to evaluate fear and anxiety felt during different situations and then rated again on a 4-point Likert scale that measures avoidance of situations (from 0 = never 0% to 3 = usually 67–100%). Higher scores reflect higher levels of social phobia [43, 44].

The SCL-90-R is a 90-items scale that evaluate psychopathologic symptoms, measuring levels of psychopathology distress in the past week. Each item is rated on a 5‐point Likert scale (from 0 = not at all to 4 = extremely), composed of 9 primary symptoms scales and 3 global indices of distress. Higher scores reflect higher levels of psychopathologic symptoms [45, 46].

### Statistical analysis

Continuous variables were reported as mean ± standard deviation, whereas categorical variables were reported as percentage. For the cross-sectional study, Analysis of Covariance (ANCOVA) was performed to compare all groups, with age and education level entered as covariates. Post-hoc paired contrasts with Tukey B test were performed for pairwise comparisons. Linear mixed models (ANOVA mixed model with random intercept) were adopted for longitudinal data, with the same covariates. In particular, these models were used to study the variation (time effect) of clinical variables within different timepoints. Longitudinal moderation analyses were performed by entering the Time*Group interaction in the models. Linear regression models were used to test the relationships between the longitudinal changes of the variables. All analyses were performed using SPSS version 27.0 [47].

Regarding the cross-sectional study, small differences between groups in terms of AQ scores were expected. Consequently, power analysis indicated that a total sample of at least 560 participants (140 per group) was needed to detect an effect size corresponding to a partial η2 of as little as 0.03 with a power of 0.95 (*ɑ* = 0.05), for an ANCOVA with four groups and up to two covariates. For the prospective study, 53 participants were needed to detect the same effect size for a repeated-measures ANOVA (within factor), with the same type I error rate, a power of 0.90, and a correlation among repeated measures of at least 0.70. Power analysis was performed using G*Power [48].

## Results

### Sociodemographic differences between transgender and cisgender people

A total of 789 subjects were considered for the cross-sectional study, including 401 cisgender persons (*n* = 229 cismen and *n* = 172 ciswomen) and 388 transgender persons (*n* = 206 transmen and *n* = 182 transwomen).

The sociodemographic and clinical characteristics of the samples included are summarized in Table 1. No differences in terms of age were observed between cisgender people and transgender people (*p* = 0.25). Cisgender people showed significant higher educational level compared to transgender groups (*p* < 0.001). Furthermore, transgender groups were more commonly non-Italian natives than cisgender ones (*p* < 0.001). In addition, medication use was more frequently reported in transgender individuals compared to cisgender ones (*p* < 0.001). Considering psychological/psychotherapeutic paths, cisgender people reported to have asked for a psychological/psychotherapeutic support more frequently than transgender people (*p* < 0.001). Thus, all the following results have been adjusted for age and years of school.

**Table 1:** Sociodemographic characteristics between transgender and cisgender people

	Cismen (42.9%, *n* = 229) % (*n*)	Ciswomen (57.1%, *n* = 172) % (*n*)	Transmen (53.1%, *n* = 206) % (N)	Transwomen (46.9%, *n* = 182) % (*n*)	*p* value
Mean age	26.63 ± 5.58	26.44 ± 7.07	27.22 ± 8.26	27.86 ± 8.24	0.25
Educational level	13.90 ± 2.63	13.39 ± 2.79	**11.27 ± 3.76**	**11.28 ± 3.83**	** < 0.001**
Non-Italian natives	8.6% (*n* = 3 )	2.9% (*n* = 1)	**37.1% (*n* = 13)**	**51.4% (*n* = 18)**	** < 0.001**
Medication use	12.9% (*n* = 18)	23.0% (*n* = 32)	**35.3% (*n* = 49)**	**28.8% (*n* = 40)**	** < 0.001**
Psychological/psychotherapeutic support	34.3% (*n* = 23)	26.9% (*n* = 18)	**19.4% (*n* = 13)**	**19.4% (*n* = 13)**	** < 0.001**

Boldfaced numbers highlight statistically significant differences between groups

### Differences between groups in terms of autistic traits and psychological functioning

**Fig. 1:**
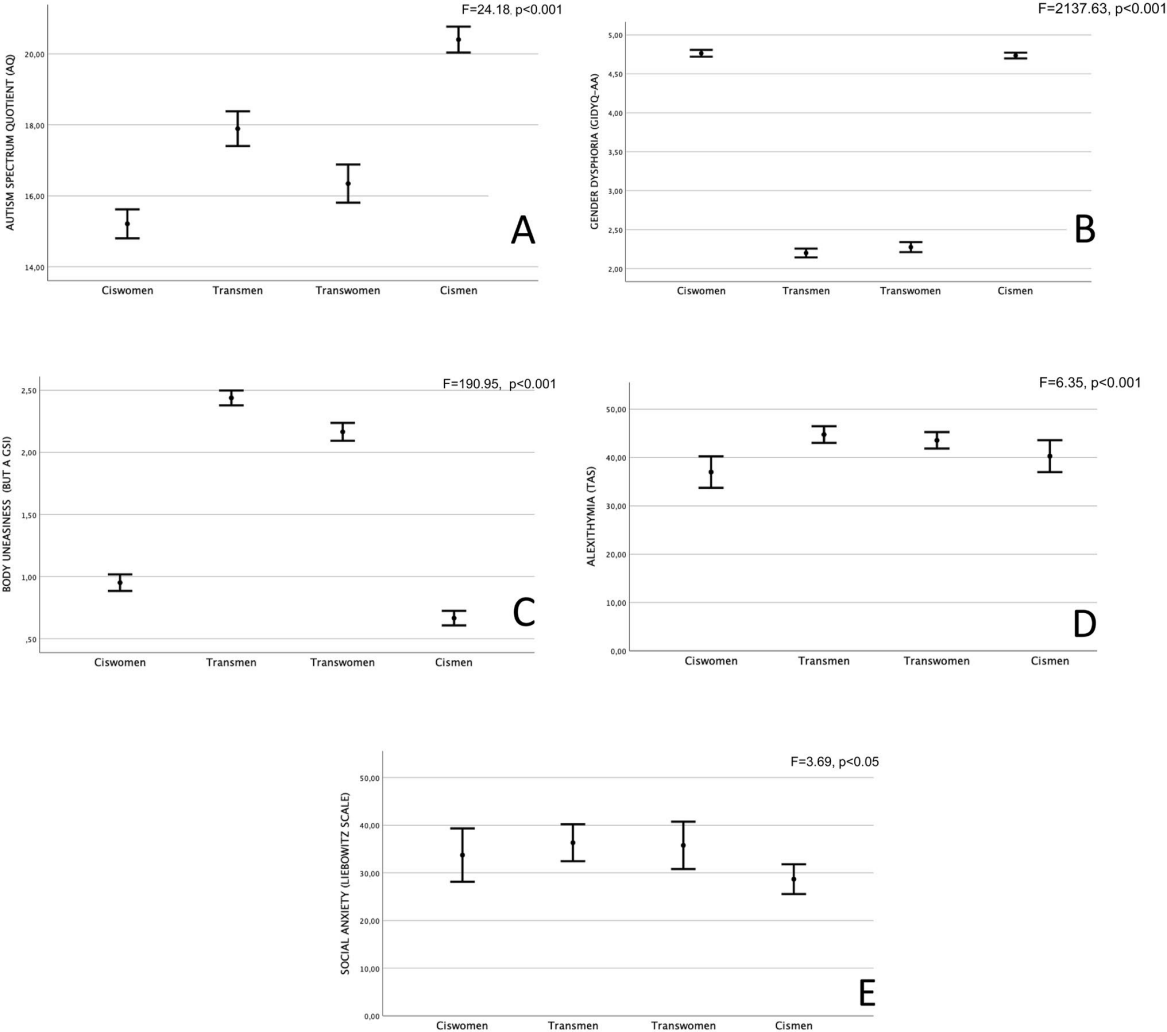
Psychological differences between transgender and cisgender people. The figure shows differences in terms of autistic traits (Fig. 1a), gender dysphoria (Fig. 1b), body uneasiness (Fig. 1c), alexithymia (Fig. 1d) and social anxiety (Fig. 1e)

Groups showed significant differences in terms of autistic traits according to AQ scores. In particular, ciswomen showed significant lower scores of AQ compared to all other groups, while cismen reported significant higher scores of AQ than all other groups (16.26 ± 5.97 and 15.59 ± 5.41 for transwomen and ciswomen, respectively; 17.38 ± 6.67 and 20.67 ± 5.41 for transmen and cismen, respectively; Fig. 1a). Other differences between groups in AQ subscales are reported in Table 2.

**Table 2:** Differences between groups in terms of autistic traits and psychological functioning

	Cismen (42.9%, *n* = 229)	Ciswomen (57.1%, *n* = 172)	Transmen (53.1%, *n* = 206)	Transwomen (46.9%, *n* = 182)	F	*p* value
AQ social skills	3.23 ± 1.83^d^	2.08 ± 1.74^b^	3.23 ± 2.63	2.48 ± 2.04	11.21	< 0.001
AQ imagination	4.32 ± 1.74^c^	2.74 ± 1.78^e^	3.13 ± 1.92	3.06 ± 1.69^a^	27.07	< 0.001
AQ attention switching	3.94 ± 1.80	3.64 ± 1.81^b^	4.33 ± 2.15^b^	4.02 ± 2.08	2.85	*p* < 0.05
AQ attention to detail	5.65 ± 2.06^f^	5.26 ± 2.07^b^	4.59 ± 2.18	4.97 ± 2.08	7.00	< 0.001
AQ communication	4.13 ± 1.97^c^	2.37 ± 1.87	2.60 ± 2.10	2.20 ± 2.04	32.60	< 0.001
GIDYQ-AA Subjective GD subscale	4.85 ± 0.26	4.89 ± 0.18	1.96 ± 0.40^a^	1.95 ± 0.36^a^	2610.86	< 0.001
GIDYQ-AA Social subscale	4.46 ± 0.22	4.45 ± 0.22	2.75 ± 0.57^a^	2.87 ± 0.69^a^	370.92	< 0.001
GIDYQ-AA Somatic subscale	4.94 ± 0.27	4.99 ± 0.35	1.36 ± 0.67^a^	1.52 ± 0.71^a^	1482.01	< 0.001
GIDYQ-AA Legal subscale	4.96 ± 0.25	4.96 ± 0.24	2.59 ± 0.87^a^	2.67 ± 0.98^a^	334.51	< 0.001
BUT avoidance	0.33 ± 0.81	0.38 ± 0.68	2.11 ± 1.10^a^	1.70 ± 1.10^a^	110.82	< 0.001
BUT compulsive self-monitoring	0.67 ± 0.92	0.86 ± 0.78	1.27 ± 0.80^a^	1.87 ± 1.01^a^	42.13	< 0.001
BUT depersonalization	0.31 ± 0.78	0.29 ± 0.54	2.67 ± 0.92^a^	2.27 ± 1.10^a^	254.34	< 0.001
BUT body image concerns	0.86 ± 1.02	1.14 ± 0.95	3.59 ± 0.88^a^	3.01 ± 1.10^a^	252.59	< 0.001
BUT weight phobia	0.97 ± 1.07	1.39 ± 1.04	2.61 ± 0.98^a^	2.50 ± 0.96^a^	93.41	< 0.001
BUT positive symptom distress index	1.60 ± 0.83	1.91 ± 0.70	2.90 ± 0.70^a^	3.07 ± 0.70^a^	68.39	< 0.001
BUT positive symptom total	6.60 ± 6.49	10.75 ± 7.14	17.68 ± 8.53^a^	22.22 ± 9.23^a^	49.32	< 0.001
Liebowitz fear/anxiety subscale	16.21 ± 10.61	18.37 ± 14.20	21.01 ± 15.31^a^	20.54 ± 16.44^a^	4.54	< 0.005
Liebowitz avoidance subscale	12.98 ± 10.55	13.83 ± 12.09	17.02 ± 13.81^a^	17.64 ± 15.16^a^	3.76	0.011

*AQ* Autism spectrum quotient, *GIDYQ-AA* gender identity/gender dysphoria questionnaire for adolescents and adults, *GD* gender dysphoria, *BUT* body uneasiness test, *LSAS* Liebowitz social anxiety scale

^a^Transwomen and transmen vs. ciswomen and cismen, ^b^ciswomen vs. transmen, ^c^cismen vs. other groups, ^d^Cismen vs. ciswomen, ^e^Ciswomen vs. transmen and cismen, ^f^Cismen vs. transmen and transwomen

Transgender people showed significantly lower GIDYQ-AA scores, indicating higher levels of GD when compared to cisgender ones (2.27 ± 0.38, 2.20 ± 0.39, 4.76 ± 0.12 and 4.74 ± 0.19, respectively, for transwomen, transmen, ciswomen, and cismen; Fig. 1b). A similar pattern was observed for all GIDYQ-AA subscales (Table 2). Furthermore, when compared to the rest of the sample, both transwomen and transmen showed significantly higher body uneasiness levels, according to BUT GSI scores (2.35 ± 0.87, 2.56 ± 0.74, 0.95 ± 0.74 and 0.67 ± 0.80 for transwomen, transmen, ciswomen and cismen, respectively; Fig. 1c). Similar results were observed for BUT subscales, with significant higher scores in transgender groups compared to cisgender ones (all *p* < 0.001; Table 2).

Considering alexithymia (TAS), transgender individuals scored significantly higher when compared to cisgender ones (43.88 ± 12.12, 45.51 ± 12.35, 37.17 ± 10.68 and 40.03 ± 10.05 for transwomen, transmen, ciswomen, and cismen, respectively; Fig. 1d). In addition, both transwomen and transmen showed significant higher social anxiety levels (LSAS) when compared to other groups (35.94 ± 28.51, 37.52 ± 27.58, 28.79 ± 19.80 and 33.84 ± 25.07 for transwomen, transmen, ciswomen and cismen, respectively, Fig. 1e). A similar figure was observed for LSAS fear and avoidance subscales (Table 2). Finally, no significant differences were found between groups (*p* = 0.61) in terms of general psychopathology, according to Global Severity Index (SCL-90R GSI).

### Follow-up data

**Fig. 2:**
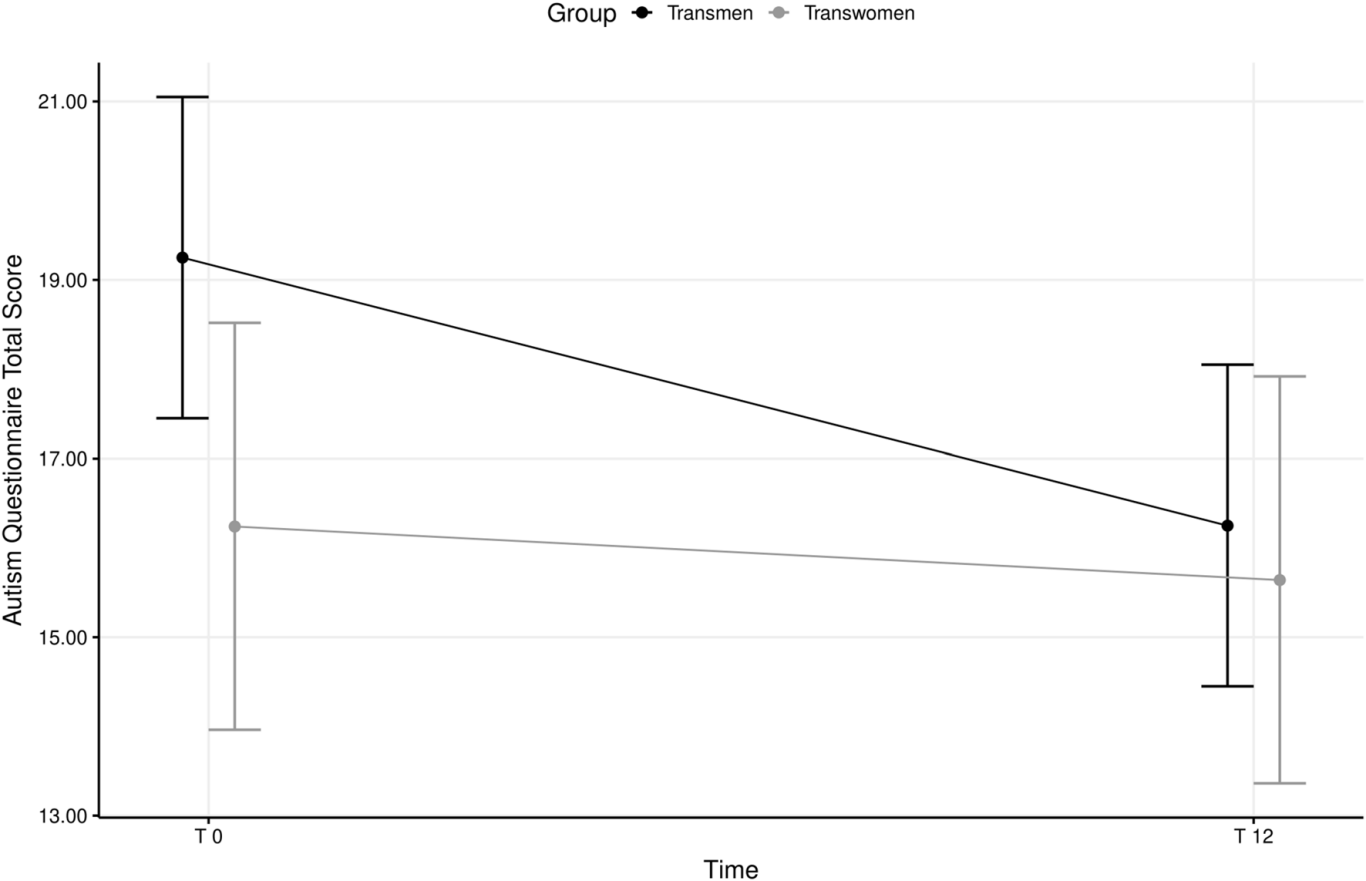
Longitudinal trend of autistic traits during gender affirming hormonal treatment

Longitudinal data of the subsample evaluated at the 12-month follow-up are reported in Table 3. After GAHT, study participants showed a significant reduction in subjective GD (as indicated by higher scores in the respective GIDYQ-AA subscale), whereas social and socio-legal domains worsened (Table 3). A significant improvement in both general psychopathology and body uneasiness levels was also observed (Table 3). As for autistic traits, AQ scores decreased significantly after 12 months of treatment (Fig. 2). A similar pattern was observed for AQ domains related to attention switching and communication skills (Table 3). Alexithymia and social anxiety also significantly decreased after GAHT (Table 3); however, these ameliorations could not have mediated that of autistic traits, given that the variations of LSAS and TAS over time did not predict the change in AQ scores (b_LSAS_ = 0.02, *p* = 0.299; b_TAS_ = 0.72, *p* = 0.209).

**Table 3:** Longitudinal trend of psychometric measures after 12 months of gender affirming hormonal therapy

	Baseline (T0)	Follow-up (T12)	Time effect
AQ_total score	18.06 ± 6.25	16.02 ± 5.19	− 2.08***
AQ_social skill	3.21 ± 2.40	2.94 ± 2.27	− 0.28
AQ_attention switching	4.50 ± 2.14	3.82 ± 2.23	− 0.69**
AQ_attention to detail	5.09 ± 2.17	4.77 ± 2.18	− 0.32
AQ_communication	2.50 ± 1.65	1.89 ± 1.33	− 0.62**
AQ_imagination	3.29 ± 1.82	3.12 ± 1.46	− 0.17
GIDYQ-AA total score	2.19 ± 0.29	2.05 ± 0.25	− 0.15***
GIDYQ-AA subjective GD	1.90 ± 0.25	2.00 ± 0.31	0.10*
GIDYQ-AA social	2.77 ± 0.54	2.42 ± 0.42	− 0.36***
GIDYQ-AA somatic	1.33 ± 0.57	1.26 ± 0.45	− 0.05
GIDYQ-AA sociolegal	2.77 ± 0.78	1.87 ± 1.00	− 0.92***
BUT-A GSI	2.39 ± 0.87	1.49 ± 0.83	− 0.94***
LSAS total score	41.17 ± 27.59	27.98 ± 30.49	− 12.47**
TAS total score	44.47 ± 11.64	40.89 ± 10.34	− 3.63*
SCL-90-R GSI	0.67 ± 0.51	0.40 ± 0.37	− 0.29***

Statistical analyses were adjusted for age and years of school *p < 0.05; **p < 0.01; ***p < 0.001

Mixed-model moderation analysis for AQ Total Score indicated a significant Time*Group interaction term (intercept = 19.08, *p* < 0.001; b_Time_ =  − 3.00, *p* < 0.001; b_Transwomen_ =  − 3.00, *p* = 0.047; b_Time*Transwomen_ = 2.40, *p* = 0.049), indicating that the improvement in autistic traits was significant only for transmen, whose baseline scores were higher than those of transwomen, while they were similar at follow-up (*p* = 0.683) (Fig. 2). A similar trend was observed for the communication subscale (intercept = 3.51, *p* < 0.001; b_Time_ =  − 0.95, *p* < 0.001; b_Transwomen_ =  − 0.68, *p* = 0.078; b_Time*Transwomen_ = 0.87, *p* = 0.025).

## Discussion

This study investigated the meaning of autistic traits in adults with GD, including data on the longitudinal trend during GAHT. The main findings were as follows: (i) there was a gradient in which cisgender women reported the lowest autistic trait scores, cisgender men the highest, and transgender groups intermediate scores, with transmen scoring higher than transwomen; (ii) over the course of the first 12 months of GAHT, there was a significant reduction in AQ scores, particularly in attention switching and communications skills domains. Moderation analysis showed that these changes happened only in transmen, whose AQ levels were comparable to those of transwomen after GAHT; (iii) alexithymia and social anxiety also decreased at follow-up; however, there was no association between their variations and that of autistic traits.

Autistic traits in transgender people have been the focus of several studies. Furthermore, the nature of their association with GD has been a matter of debate in recent years [35, 36, 48]. The higher levels of autistic traits found in transmen with respect to transwomen is apparently in line with the EMB theory [28]. However, the fact that the transgender groups significantly differed from both ciswomen and cismen is not. Moreover, their intermediate scores between cis groups do not agree with the supposed link between ASD and GD [49], especially if we take into consideration that the average observed scores in all groups were far from being above the clinical cutoff of 32 + identified in the original validation study [37].

The longitudinal findings confirm the efficacy of GAHT in reducing subjective GD and other psychological distress domains, such as body uneasiness [50–56]. Furthermore, the normalization of AQ scores following GAHT, after which the two trans groups showed comparable levels, suggests that in this population autistic traits measured by AQ scores could represent a GD-associated state, which subsides with GAHT just like other indices of distress, such as general psychopathology and social anxiety. From this perspective, the autistic traits measured in our sample, changing with GAHT, could be explained by other psychopathological constructs (as anxiety disorders associated with GD, avoidant and obsessive personality traits, discrimination and stigma).

These findings directly contradict those of Nobili and Colleagues (2020) who instead observed that autistic traits remained stable after 1 year of GAHT in a sample of transgender adults [5]. Overall, the present results seem to be more in line with the theory stating that autistic traits in transgender people may not represent ASD per se, but could instead reflect social deficits following the social deprivation that is often observed [35, 36]. Moreover, Turban predicted that these “traits” are probably reversible with improved social engagement which is likely a consequence of a gender affirming path [36] and could represent the cause of the longitudinal trend observed in the present study.

In this context, it is critical to note that AQ showed less accuracy in predicting autistic traits in clinical populations, especially in the presence of psychiatric comorbidities [57]. From this perspective, the fact that its scores could represent a measurement of general social distress in transgender people does not seem too far-fetched. Finally, since it was observed only in the transmen group, the hypothesis that the decrease in AQ scores is a consequence of the known effects of testosterone on neuronal activation and cognitive performance [29, 30] cannot be ruled out now.

The decrease in social anxiety and alexithymia levels has been proposed as a possible mediator of the decrease in the levels of these autistic traits; however, the absence of association between the longitudinal variations of these measures led to the exclusion of this hypothesis. Regarding social anxiety, it should be noted that the LSAS was developed for assessing fear and avoidance due to social phobia, and in social contexts that are relevant to people with social anxiety disorder. Consequently, this questionnaire may not be able to properly capture the components of social distress that are associated with GD, given that they are not due to social phobia but rather to anti-trans stigma. [58].

### Limitations

The results of our study should be interpreted in the light of the following limitations. First of all, given the small sample size of the prospective study (*n* = 62), our data should be considered as preliminary. Furthermore, for ethical issues, we did not perform a randomized, placebo-controlled study (with a no-GAHT control group). Moreover, all the questionnaires used in this study were self-administered. In addition, we enrolled a study population of transgender people referring to Italian Gender Clinics, so the results may not be generalizable to all transgender population. Furthermore, future researches should consider other psychopathological constructs—as anxiety disorders associated with GD, personality traits (as avoidant and obsessive), discrimination and stigma—to better understand the association between GD and autistic traits. Moreover, presence of organic condition impacting on the testosterone levels should be included among the exclusion criteria. Finally, 12-month follow-up data are lacking.

## Conclusions

The results of our study suggest that the autistic traits measured in our sample may represent an epiphenomenon of GD rather than being part of an ASD condition, since they significantly decreased after 12 months of GAHT. However, further longitudinal studies are needed on this topic to unravel the complex relationship between autistic-like symptomatology and GD, with a focus on the possible mediators and moderators of variations over time. Since these “autistic” traits could mask more complex and deeper distress dimensions of GD, a proper assessment could be critical to correctly identify and target them during gender affirming path in the clinical setting.
